# Effects of physical activity as an adjunct treatment on healing outcomes and recurrence of venous leg ulcers: A scoping review

**DOI:** 10.1111/wrr.12995

**Published:** 2022-02-10

**Authors:** Yunjing Qiu, Christian R. Osadnik, Victoria Team, Carolina D. Weller

**Affiliations:** ^1^ School of Nursing and Midwifery Monash University Melbourne Victoria; ^2^ Department of Physiotherapy Monash University Frankston Victoria Australia

**Keywords:** adjunct treatment, compression, healing, physical activity, recurrence, venous leg ulcer

## Abstract

Healing time is protracted and ulcer recurrence is common in patients with venous leg ulcers. Although compression is the mainstay treatment, many patients do not heal timely. Physical activity may be a clinically effective adjunct treatment to compression to improve healing outcomes. This scoping review provides a broad overview of the effect of physical activity as an adjunct treatment to compression on wound healing and recurrence. We followed the six‐step framework developed by Arksey and O'Malley. We searched electronic databases and trial registration websites for relevant studies and ongoing trials. Two authors independently screened and selected articles. Findings were presented in a descriptive statistical narrative summary. We consulted and presented our findings to the wound consumer group to ensure the relevance of our study. Physical activity interventions in 12 out of the 16 eligible studies consisted of only one component, eight studies were resistance exercises, three studies reported ankle and/or foot range of motion exercises, and one study reported aerobic/walking exercises. The remaining four studies involved multicomponent exercise interventions. Resistance exercise combined with ankle and/or foot range of motion exercise minimised ulcer size on day 12 (intervention group: 4.55 ± 1.14 cm^2^ vs. control group: 7.43 ± 0.56 cm^2^) and improved calf muscle pump performance on day 8 (ejection fraction: 40%–65%; residual volume fraction: 56%–40%). We identified one study that reported ulcer recurrence rate with no clinical difference in the intervention group versus the control group (i.e., 12% in intervention vs. 5% in control). Our review identified that resistance exercise was the most common type of physical activity intervention trialled in the published literature. Resistance exercise combined with ankle and/or foot range of motion exercise appears to be effective adjunct treatments; however, the overall evidence is still relatively weak as most programmes had a short intervention period which limited clinical outcomes.

**Abbreviations**
ANZCTR: Australian New Zealand Clinical Trials Registry
BMI: body mass index
CENTRAL: The Cochrane Central Register of Controlled Trials
CERT: Exercise Reporting Template tool
EQ‐5D‐5L: EuroQol‐five dimensions five level version questionnaire
JBI: Joanna Briggs Institute
PA: physical activity
PRISMA: The Preferred Reporting Items for Systematic Reviews and Meta‐Analyses extension
QoL: quality of life
RCT: randomised control trial
SF‐12: 12‐item short form survey
SF‐18: 18‐item short form survey
SR: systematic review
VEINES‐QOL: Venous Insufficiency Epidemiological and Economic Study‐quality of life
VLU: venous leg ulcer

## 1 BACKGROUND

Venous leg ulceration (VLU) is commonly caused by chronic venous insufficiency which causes hypertension in the lower limbs.^1^ The prevalence of VLU is estimated to be 1% of adults worldwide^2^, ^3^; however, this figure rises with age, reaching 1.69%–2% among the population aged 65 and over.^4^, ^5^

Current best clinical practice is application and adherence to compression bandages or stockings to reduce the hydrostatic pressure in the lower legs and aid venous return.^6^, ^7^ Healing rates vary from 17%–67% and the healing period ranges from 45 to 112 days.^8^, ^9^, ^10^ Further, up to one third of VLUs reoccur when treated with compression therapy alone.^11^ The recurrence rate in the first three months post healing has been reported at 36%,^12^ and more than one in four patients experience multiple episodes of recurrent ulceration and recurrence in their lifetime.^13^ VLUs cause physical pain, discomfort and mobility issues.^14^, ^15^ Repeated cycles of VLU healing and recurrence cause emotional distress and lead to poor quality of life (QoL).^16^, ^17^ Adjunct physical activity (PA) treatments, such as exercise, may improve healing rates and reduce VLU recurrence when used in conjunction with compression therapy.^18^, ^19^

Deterioration of calf muscle pump function in patients with VLUs is reported to be associated with delayed healing.^20^ Past evidence identified that PA stimulates the calf muscle pump function, and this increases venous pressure, promotes blood return and subsequently improves ulcer healing.^21^ Previous reviews have examined various types of PA interventions as adjunct treatments to compression therapy for VLU management, including (progressive) resistance exercise, aerobic exercise, walking and ankle exercise.^18^, ^22^, ^23^ Although these reviews indicate that PA interventions improve calf muscle pump function, it does not define which type of PA may increase calf muscle pump function.^18^, ^22^, ^23^ One review suggests that progressive resistance and aerobic exercise would improve ulcer healing.^18^ This review, however, does not clarify how the exercise intervention improves ulcer healing, whether it accelerates ulcer healing or reduces ulcer size.^18^ The current clinical practice guidelines also state that PA interventions aim to enhance calf muscle strength, and recommend to incorporate exercise into VLUs management plan.^24^, ^25^, ^26^, ^27^ Nevertheless, the type of PA intervention improving ulcer healing remains unknown, and the evidence base for this recommendation, as acknowledged by the guideline authors, is very limited.^24^, ^25^, ^26^, ^27^ We sought to scope and synthesise available evidence to better understand the types of PA that have been studied, as well as the effects of PA as an adjunct treatment to complement compression therapy in people with VLUs.

## 2 METHODS

This scoping review was guided by the six‐step framework developed by Arksey and O'Malley^28^ and updated by Levac et al.^29^ and the Joanna Briggs Institute (JBI).^30^ The Preferred Reporting Items for Systematic Reviews and Meta‐Analyses (PRISMA)^31^ extension for scoping reviews checklist was also followed to ensure all essential components have been covered. The protocol for this review has been published in July 2021.^32^

### 2.1 Stage 1: Identifying the research question

The scoping review aimed to provide a comprehensive overview of the effect of PA as an adjunct treatment to compression therapy on the healing and recurrence of VLUs. To address this aim, the following questions were identified.

1. What types of adjunct PA/exercise interventions have been examined as adjunct treatments for management of VLUs?
2. What methods (i.e., instruments/metrics) have been used to evaluate the key outcomes of interest regarding PA/exercise interventions applied for people with VLUs?
3. What is the effect of PA/exercise interventions on patient/clinical outcomes?

### 2.2 Stage 2: Identifying relevant studies

This scoping review followed the three‐step search strategy guided by JBI^30^ to identify relevant studies. The following electronic databases were search without data limits:

- Ovid Medline (1946 to 24 October 2020);
- Ovid CINAHL (1982 to 26 October 2020);
- The Cochrane Central Register of Controlled Trials (CENTRAL) (26 October 2020).

The research team developed search strategies with assistance from an experienced librarian. The full search strategy used for these databases can be found in Supporting Information S2. Search terms incorporated the use of wildcards and Boolean operators where appropriate and the combination of both keywords and controlled vocabulary specific to individual databases. Reference lists of all eligible studies were screened for any additional studies that were not identified via the electronic database search. We also searched ICTRP WHO (https://apps.who.int/trialsearch/), Clinical Trials.gov (https://clinicaltrials.gov/) and Australian New Zealand Clinical Trials Registry (ANZCTR) (http://www.anzctr.org.au/) for ongoing clinical trials. Grey literature was searched via inspecting the first five pages of Google Scholar search results, using the terms ‘venous leg ulcer and physical activity’ and ‘venous leg ulcer and exercise’. The inclusion and exclusion criteria were designed to be highly inclusive (Table 1).

**TABLE 1:** Inclusion and exclusion criteria

	Inclusion criteria	Exclusion criteria
Participants	Adults (18 years and over) with a clinically diagnosed VLU	People with other types of ulcers (i.e., arterial ulcers, ulcers with multiple aetiology)
Concept	Studies focus on PA as an adjuvant treatment to compression therapy Physical activities include recreational activities, education programmes, exercises or any combination of these Compression therapies include compression bandages, compression stockings, or any combination of compression therapies	Studies that do not investigate the effect of PA as an adjunct treatment to compression on VLU healing and recurrence
Context	Studies conducted in any setting (i.e., hospital, community, home or residential area)	Studies published in languages other than English
Study types	Experimental and quasi‐experimental study designs (i.e., RCT, non‐equivalent group designs, pre‐test and post‐test studies, interrupted time‐series studies) Observational studies (i.e., prospective/retrospective cohorts, cross‐sectional studies, case series studies)	Qualitative studies Literature reviews, opinion text, oral presentations, conference notes, abstracts were excluded Studies that are unable to retrieve full‐text articles
Outcomes	Studies that have reported one or more of the following outcomes: Primary outcomes: time to healing; proportion of ulcers healed during the trial period; rate of changes in the area of the ulcer during the trial period; incidence of recurrence of healed VLUs Secondary outcomes: changes in calf muscle pump function; quality of life; wound pain; adverse events; economic outcomes (patient or healthcare perspective)	Studies that have not reported any outcomes of interest

Abbreviations: PA, physical activity; RCT, randomised control trial; VLU, venous leg ulcer.

### 2.3 Stage 3: Study selection

Citations from each database were uploaded into EndNote, de‐duplicated using default settings and exported to Covidence^33^ for screening. Two review authors independently screened the titles and abstracts of all identified studies and rated them as exclude, include or unclear. All citations rated as include or unclear progressed to full text review by two independent review authors. Any disagreements at any stage of screening were resolved via discussion and mutual agreement or via assistance of a third independent assessor.

### 2.4 Stage 4: Charting the data

Data from each included study were extracted by one author independently using data extraction tables created and piloted prior to the study by two authors. All extracted data were checked for accuracy by a second author, with any discrepancies resolved via discussion. Where available, the following data were extracted from each included study:

- Characteristics of studies
- Population characteristics
- PA/exercise interventions characteristics
- Primary outcomes, including time to heal, proportion of ulcers healed during the trial period, rate of changes in the area of the ulcer during the trial period and incidence of recurrence of healed VLUs
- Secondary outcomes, including changes in calf muscle pump function, QoL, pain, adverse events and economic outcomes. Adverse events were defined as any unfavourable sign, symptom or occurrence that occurred during treatment that may or may not have been related to treatment.^34^ These included, but were not limited to, increased exudate from ulcer sites, infection or hospitalisation.

### 2.5 Stage 5: Collating, summarising and reporting results

The findings of this review were reported via descriptive analysis and a narrative summary of findings using text and tabulations. We reported the characteristics of the PA interventions against the Consensus on Exercise Reporting Template (CERT) criteria.^35^ This instrument details the core elements that must be provided in order to ensure adequate reporting of such interventions for the purposes of critical appraisal and replication.

This review classified PA/exercise interventions into the following three categories based on their features:

1. Resistance exercise (non‐progressive and progressive): This included interventions involving repeated limb movement against a resistance exerted via exercise equipment (e.g., elastic resistance band, resistance pedal ergometer) or body weight (e.g., weight bearing heel raises) aiming to improve aspects of strength.
2. Aerobic exercise/walking: Aerobic exercise included cycling and walking interventions that involved a clearly defined frequency, intensity, type and/or timing (duration) that aimed to enhance blood circulation by increasing one's heart rate.^36^ In contrast, walking interventions that involved daily step count targets (e.g., 10,000 steps/day goal) but lacked details regarding bout length (i.e., how long participants were to walk for at any given time) were classified as ‘walking’.
3. Ankle/foot range of motion exercise: This included interventions involving repeated movement of the foot/ankle joint (e.g., dorsiflexion, plantarflexion, ankle rotations) targeting restoration of ankle mobility and/or the promotion of blood flow via the foot/ankle pump.

The effectiveness of each exercise component on the outcomes of interest was reported in the review. Individual studies were allowed to be included in multiple categories in the case of multicomponent PA/exercise interventions.

### 2.6 Stage 6: Consultation

The research team consulted an established external wound consumer advisory group. The goal of this consultation was to gain insights from diverse viewpoints with unique knowledge from people who live with VLUs to help us define the study objective and ensure that the findings were relevant and meaningful from a patient perspective. During the first consultation, the members of the wound consumer advisory group identified what they would like to know about how different types of PA may aid ulcer healing. This suggestion aligned with the research questions of this review. We presented a summary of the review findings to the wound consumer advisory group members, and asked the group to provide their comments in regards to clarity of the included information. We also asked the group to identify any additional issues relating to PA as adjunct treatment to VLU management that were not addressed in the review findings to ensure the relevance of our findings to this group.

## 3 RESULTS

### 3.1 Study selection

**FIGURE 1:**
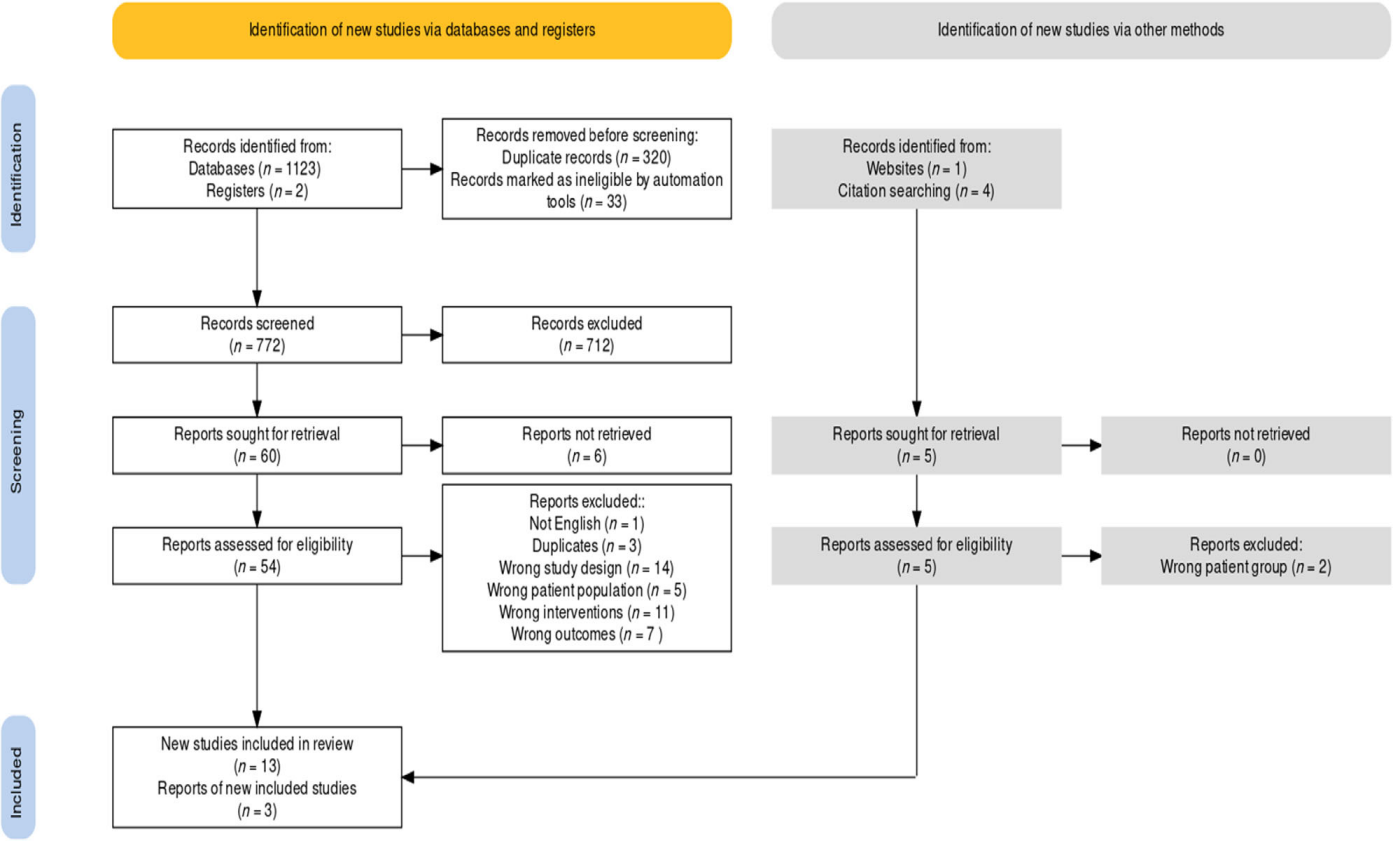
Study selection flowchart [Color figure can be viewed at wileyonlinelibrary.com]

The search yielded a total of 1130 studies. After removing duplicates and checking against inclusion criteria, 59 articles were eligible for full‐text review and 16 studies met the selection criteria and were included in the final analysis. Reasons for full‐text exclusion are presented in a PRISMA flow chart (Figure 1).

### 3.2 Study characteristics

Detailed characteristics of each eligible study are presented in the Supporting Information S3. Included studies were published between March 1999 and February 2021, with 71% (*n* = 12) published after 2012. Thirteen studies were randomised control trials (RCTs), two were single‐armed cohort studies, one was a prospective controlled study. Intervention duration ranged from 7 days to 52 weeks, with 12 weeks being the most common (*n* = 10). The sample size of RCTs varied from 11 participants to 224 participants per study.

### 3.3 Participants

Most participants in the included studies were over 60 years old and 330 of 522 (63.2%) were female. Five studies reported the body mass index (BMI), and the mean BMI of participants were 30 kg/m^2^ or above.^22^, ^24^, ^37^, ^38^, ^39^ More than 80% (*n* = 319) of participants had VLUs for over 3 months, and the mean/median size of the ulcers in most participants was less than 10 cm^2.^ ^37^, ^38^, ^39^, ^40^, ^41^, ^42^, ^43^, ^44^ Intervention groups and control groups were comparable across all included studies regarding ulcer number, ulcer duration, ulcer size and mobility level. Details of baseline participants characteristics are summarised in Supporting Information S4.

### 3.4 Types of adjunct PA interventions used for VLU management

Out of 16 studies, 10 studies evaluated the effect of PA/exercise interventions involving single‐component exercise interventions (i.e., resistance exercise, ankle/foot range of motion exercise),^37^, ^38^, ^42^, ^43^, ^45^, ^46^, ^47^, ^48^, ^49^, ^50^ and five studies reported PA/exercise interventions involving multicomponent exercise interventions (i.e., resistance exercise plus aerobic exercise/walking and/or ankle/foot range of motion exercise).^39^, ^40^, ^41^, ^44^, ^51^ A summary of exercise components involved in PA/exercise interventions in eligible studies is outlined in Table 2.

**TABLE 2:** A summary of exercise components involved in PA/exercise interventions in eligible studies

Author (year)	Resistance exercise, non‐progressive	Resistance exercise with progressive overload	Aerobic exercise/walking	Ankle/foot range of motion exercise	Combination therapy
Yang et al. (1999)^47^	N	Y (ET)	N	N	N
Kan and Delis (2001)^43^	N	Y (ET)	N	N	N
Davies et al. (2007)^41^	N	Y (ET)	N	Y (ankle rotation)	Y
Jull et al. (2009)^42^	N	Y (ET)	N	N	N
Meagher et al. (2012)^48^	N	N	Y (walking—step‐targeted)	N	N
Ahmed et al. (2013)^40^	Y (ST)	N	N	Y (DF)	Y
O'Brien et al. (2013)^38^	N	Y (ET)	N	N	N
Sallam et al. (2017)^39^	N	Y (ET)	N	Y (DF)	Y
O'Brien et al. (2017)^44^	N	Y (ET)	Y (walking‐aerobic)	N	Y
Domingues et al. (2018)^49^	N	N	N	Y	N
Klonizakis et al. (2018)^51^	N	Y (ST)	Y (treadmill walking, cycling‐aerobic)	N	Y
Mutlak et al. (2018)^45^	N	N	N	Y (DF)	N
Nabil et al. (2019)^50^	N	Y (ET)	N	N	N
Jonker et al. (2020)^37^	Y (ST)	N	N	N	N
Kelechi et al. (2020)^46^	N	N	N	Y (DF; PF; ankle rotation; toe and foot taps; lower extremity kickouts)	N
Ongoing studies					
Herraiz‐Ahijado and Folguera‐Álvarez (2021)^52^	NC	NC	NC	NC	NC

Abbreviations: DF, dorsiflexion; ET, endurance training; NC, not clear; PA, physical activity; PF, planter flexion; ST, strength training.

More than half (*n* = 11) of PA/exercise programmes were conducted in the home environment without supervision.^37^, ^38^, ^41^, ^42^, ^44^, ^45^, ^46^, ^47^, ^48^, ^49^, ^52^ Three studies utilised a small group format (*n* = 4 participants per group) that also involved direct supervision by a qualified exercise physiologist.^43^, ^47^, ^51^ A summary of PA interventions is presented in Table 3, with additional details (in accordance with CERT recommendations) provided in Supporting Information S5. For studies investigating supervised PA/exercise programmes, only one study reported that a specially designed report form was used to record participants' attendance for exercise sessions.^51^ By comparison, exercise diaries completed by participants themselves were commonly adopted for home‐based programmes.^41^, ^42^, ^52^ One home‐based PA/exercise programme used the ActivPal monitor at week 4 for 7 days to objectively measure participants' daily steps and compared them to self‐reported data in intervention groups.^48^ To facilitate and encourage participants to perform exercise independently at home, three studies reported the utilisation of visual tools (e.g., exercise booklets) and motivational strategies (e.g., phone calls, text messages) during trial period.^44^, ^45^, ^49^

**TABLE 3:** Description of the PA/exercise interventions for included studies following CERT

Author (year)	Exercise interventions description (sets, repetitions, duration, intensity)
Yang et al. (1999)^47^	Six weeks progressive resistance exercise programme. The exercise was performed on alternative days. Warm‐up and cool down: walking and calf stretch for 5–8 min
	Participants performed tip‐toe exercise in the standing position with their feet on the edge of a 5‐cm high step, and their hand held a rail or back of chair
	First 3 weeks: half of individual's maximum number of tip‐toe exercises ×3 reps Second 3 weeks: increased to maximum number that individual can perform ×3 reps 3–5 min rest between each rep
Kan and Delis (2001)^43^	Seven days progressive isotonic calf muscle resistance exercise, using a 4‐kg resistance pedal ergometer
	Participants performed active plantar flexion against a 4‐kg resistance in the seated position for 6 min
	First 3 days: Each set consisted ¾ of the maximal number of foot flexions that individuals can reach initially; participants need to perform this resistance exercise 3 sets × daily (6 min each set at the rate of 1 flexion per second) Last 4 days: increasing to 360 flexions × 3 sets × daily 5‐min rest between each set
Davies et al. (2007)^41^	24 weeks progressive resistance exercise programme. Three times per week, 5–10 min each time
	Warm up and cool down: ankle circling
	Plantar flexions: participants performed seated plantarflexions against an elastic band that was held at the point of offering mild resistance. They performed 15–25 reps Dorsiflexion: participants performed dorsiflexion stretches three times in the seated position for 10s without the elastic bands
	Participants' walking gait were also assessed and corrected Participants were recommended to do ankle rotations during the day
Jull et al. (2009)^42^	12 weeks progressive resistance exercises Warm up: walking for 3–5 min
	Participants performed three sets of heel raises in the standing position daily. Nurses reassessed and prescribed the number heel raises that individual need to perform at 80% of individual's maximum at baseline, 3, 6 and 9 weeks
Meagher et al. (2012)^48^	Participants were asked to take 10,000 steps per day
Ahmed et al. (2013)^40^	12 days isometric exercise, involving dorsiflexion and plantar flexion for 20 min Warm up and cool down: passive stretching of calf muscle to the point of mild tension (10 min before and 10 min after exercise)
	Participants received five exercise sessions per week, 40 min per session
O'Brien et al. (2013)^38^	12 weeks progressive resistance exercise programme Warm up and cool down: stretching calf muscle to the point of mild tension (20 s for each stretch)
	The exercise programme consisted of three stages: Stage 1: seated heel‐rises (both legs) Stage 2: standing heel‐rises (both legs) Stage 3: one‐legged heel‐rises Each stage involved four levels 10 reps × 3 sets × 3 times per day 15 reps × 3 sets × 3 times per day 20 reps × 3 sets × 3 times per day 25 reps × 3 sets × 3 times per day
Sallam et al. (2017)^39^	12 weeks progressive resistance exercise combined with foot/ankle range of motion exercise Warm up: ankle circling and plantar flexion using elastic resistance bands
	Participants performed 15–25 reps of seated plantar flexions using an elastic band to provide resistance Participants were also asked to perform dorsiflexion in the seated and standing position, starting from 30 reps and gradually increasing to 75 reps Participants performed this resistance exercise for 15 min, every second day for 12 weeks
O'Brien et al. (2017)^44^	See O'Brien (2013) Participants were also encouraged to walk for 30 min, 3 times per week
Domingues et al. (2018)^49^	12 weeks exercise targeted the lower extremities Participants repetitively moved their calves and feet for 3–4 times per day with intermittent rest
Klonizakis et al. (2018)^51^	12 weeks supervised, progressive exercise programme. Three sessions per week, 60 min per session
	Warm up: 5 min low‐intensity treadmill walking, cycling or both Cool down: 5 min low‐intensity treadmill walking or cycling; static stretch to the point of mild discomfort for 60 s
	The exercise session comprised a combination of resistance, aerobic and flexibility exercises. The intensity of each exercise was increased on individual basis once the initial goal has been achieved Aerobic exercises: participants were asked to perform either treadmill walking, cycling or both for 30 min. The speed and incline level of treadmill and resistance of bike were increased via progression Resistance exercises: four exercises were involved, two exercises targeting the thigh and hip muscles (i.e., partial squats, chair sit‐to‐stand exercise) and two exercises targeting the calf muscle (i.e., standing calf raise). Participants performed it for 15 min, with/without using dumbbells and stability balls. The weight or type of exercise were increased/changed via progression
	Participants were required to perform the exercise for 10–15 reps × 2–3 sets to the point of mild muscle fatigue
Mutlak et al. (2018)^45^	12 weeks foot/ankle range of motion exercise programme Participants performed 10 dorsiflexion each hour when participants were awake
Nabil et al. (2019)^50^	12 weeks supervised, progressive resistance exercise programme. Three sessions per week, 20 min per session
	The exercise protocol consisted of six stages: Stage 1: Seated heel raises. Stage 2: Standing heal raises. Stage 3: Seated heel raises with applying manual resistance. Stage 4: Standing heel raises with holding resistance elastic bands. Stage 5: Seated heel raises with applying resistance by weight cuffs. Stage 6: Pushing weight bar by using weight machine
	Each stage involves three levels: 10–15 reps × 3 sets × 3 times per day 15–20 reps × 3 sets × 3 times per day 20–25 reps × 3 sets × 3 times per day
Jonker et al. (2020)^37^	12 weeks plantar resistance exercise, using a 6kgs StepIt rocker pedal device
	Participants performed active plantar flexion against 6 kg resistance in the seated position for 1 min then rest for 1 min. Participants performed 10 reps × 2 sets per day at the rate of 2 s push and 2 s lift. The maximal number of pedal movements was 300 per day
Kelechi et al. (2020)^46^	6 weeks foot/ankle range of motion exercise The exercise involved toe and foot taps, plantar flexion and dorsiflexion, ankle twirls/circles and lower extremity kickouts
Ongoing studies	
Herraiz‐Ahijado and Folguera‐Álvarez (2021)^52^	6 months progressive lower limb exercise and daily walking programme
	Lower limb exercise: it comprises four exercises. Participants will perform it twice per day, 5 days/week Walking: participants will gradually increase their walking time. The targeted goal is 150 min/week (30 min/day)

Abbreviation: CERT, Exercise Reporting Template tool; reps, repetitions.

### 3.5 Outcome measures/instruments used to evaluate PA interventions in people with VLU

For outcomes used to measure VLU healing status, three studies reported time to heal,^18^, ^42^, ^51^ six studies reported proportion of ulcer healing,^37^, ^38^, ^42^, ^44^, ^48^, ^51^ and nine studies reported rate of changes in the area of ulcer^37^, ^38^, ^39^, ^40^, ^42^, ^45^, ^49^, ^50^, ^51^ during the trial period (see Supporting Information S6). One study reported ulcer recurrence rates.^51^ Calf muscle pump function were examined by three studies.^38^, ^43^, ^47^ Four studies assessed the changes in QoL.^44^, ^46^, ^49^, ^51^ Pain was reported by five studies,^37^, ^39^, ^41^, ^49^, ^51^ and six studies reported on adverse effects.^37^, ^39^, ^41^, ^42^, ^49^, ^51^ Klonizakis et al.^51^ was the only study that reported on economic outcomes both at the individual and health service levels.

The instruments for measuring ulcer healing status were diverse, including measurement sheet, ruler and digital planimetry^37^, ^42^, ^44^, ^45^, ^48^, ^52^ (see Supporting Information S3). Four studies used the same metrics (i.e., ejection fraction, residual volume fraction and instruments, and air plethysmography) to measure calf muscle pump function.^38^, ^42^, ^43^, ^47^ Five studies used visual analogue scales or numerical rating scales (e.g., 0 or 1 corresponding to no pain and 10 corresponding to the worst possible pain).^37^, ^41^, ^48^, ^49^, ^52^ Five studies reported QoL outcomes.^37^, ^44^, ^46^, ^49^, ^51^ Three of these used generic tools (i.e., SF‐18, SF‐12, FLQAw),^44^, ^46^, ^49^ one used a disease‐specific tool (Charing Cross venous ulcer questionnaire),^37^ and one incorporated both tools.^51^

### 3.6 Effectiveness of PA interventions

#### 3.6.1 Time to healing

For interventions involving resistance exercise components, two studies reported time to healing.^42^, ^51^ Although no difference was found between the intervention and the control group, a shorter ulcer healing time was reported in the control group at month 12 (intervention group: 13 weeks vs. control group: 34.7 weeks).^51^

For interventions involving aerobic exercise/walking components, two studies reported time to healing.^48^, ^51^ A clinically significant difference was reported in one study at month 12, in which the median healing time in the intervention group was less than half of that in the control group (intervention group: 13 weeks vs. control group:34.7 weeks).^51^

For interventions involving foot/ankle range of motion exercise components, no data on healing outcomes were reported.

#### 3.6.2 Proportion of ulcer healed

For interventions involving resistance exercise components, five studies measured the proportion of ulcer healed at week 12 and month 12.^37^, ^38^, ^42^, ^44^, ^51^ In comparison to control groups, a higher proportion of participants in intervention groups with ulcers healed at week 12 (intervention group: 67% vs. control group: 41%; intervention group: 50% vs. control group: 40%; intervention group:77% vs. control group: 53%) and month 12 (intervention group: 83% vs. control group: 60%).^37^, ^38^, ^44^, ^51^

For interventions involving aerobic exercise/walking components, three studies reported the proportion of ulcers healed.^44^, ^48^, ^51^ The results of three studies were similar, intervention groups had higher proportion of ulcer healed than control groups at week 12 and month 12.^44^, ^48^, ^51^

No data were reported for interventions involving foot/ankle range of motion exercise components.

#### 3.6.3 Rate of changes in the area of the ulcer

For interventions involving resistance exercise components, seven studies reported rate of changes in the area of the ulcer at week 12 and month 12.^37^, ^38^, ^39^, ^40^, ^42^, ^50^, ^51^ Two studies reported the mean ulcer size in intervention groups were smaller than control groups at the end of the trials on day 12 (intervention group: 4.55 ± 1.14 vs. control group: 7.43 ± 0.56 cm^2^; *p* ≤ 0.05) and week 12 (intervention group: 1.6 ± 0.62 cm^3^ vs. control group: 2.39 ± 0.75 cm^3^; *p* = 0.004).^40^, ^50^

For interventions involving aerobic exercise/walking components, one study reported rate of changes in the area of the ulcer at month 12. Median ulcer size in both groups was similar.^51^

For interventions involving foot/ankle range of motion exercise components, four studies reported rate of changes in ulcer area.^39^, ^40^, ^45^, ^49^ At the end of trial period, the mean ulcer size in intervention groups were smaller than control groups in all four studies.^39^, ^40^, ^45^, ^49^

#### 3.6.4 Recurrence rate

Recurrence rate was only reported by Klonizakis et al.^51^ at month 12. This study involved both resistance and aerobic exercise/walking components and found two participants (12%) in the intervention group reported ulcer recurrence in comparison with one participant (5%) in the control group.^51^

#### 3.6.5 Calf muscle pump function

Four studies examined the effects of resistance exercise on calf muscle pump function.^38^, ^42^, ^43^, ^47^ The improvements were found with respect to ejection fraction and residual volume fraction in intervention groups in all four studies.^38^, ^42^, ^43^, ^47^ The change was noticed as early as on day 8, when the ejection fraction increased from 40% to 65% (*p* = 0.006), and the residual volume fraction decreased from 56% to 40% (*p* = 0.008).^43^

No data were reported for interventions involving aerobic exercise/walking components or foot/ankle range of motion exercise components.

#### 3.6.6 Quality of life

Two studies reported participants' QoL at week 12^44^ and month 12^51^ after receiving PA interventions, respectively. These two studies involved both resistance and aerobic/walking exercise components.^44^, ^51^ One study used a generic instrument (SF‐8) to measure QoL score,^44^ while the other used both generic (EQ‐5D‐5L) and disease specific QoL instruments (VEINES‐QOL).^51^ Both studies reported that the mean or median QoL score in intervention groups were higher than control groups at week 12 (physical component score: intervention group:46 ± 10.2 vs. control group:43 ± 8.9; mental components score: intervention group: 51 [16–65] vs. control group: 51 [32–66]) and month 12 (EQ‐5D‐5L:intervention group: 0.7874 ± 0.28 vs. control group: 0.5825 ± 0.41; VEINES‐QOL: intervention group: 67.23 ± 29.86 vs. control group: 52.46 ± 34.81).^44^, ^51^

For interventions involving foot/ankle range of motion exercise components, two studies reported changes in health‐related QoL at the end of study period.^46^, ^49^ One study found a ‘significant difference’ in QoL scores between the intervention group and the control group on day 90 (*p* = 0.03); however, supportive data were not provided.^49^ One other study reported increased QoL scores in intervention group after receiving foot/ankle range of motion exercises.^46^

#### 3.6.7 Pain

For interventions involving resistance exercise components, pain scores were reported by four studies.^37^, ^39^, ^41^, ^51^ Two studies assessed pain scores at week 12,^37^, ^39^ while the others reported it at week 24^41^ and month 12,^51^ respectively. Improvements in pain were reportedly much higher in intervention groups than in control groups.^37^, ^39^

For interventions involving aerobic exercise/walking components, one study reported changes in pain scores.^51^ By month 12, mean pain score was 7.9 in the intervention group and 30.5 in the control group.^51^ However, this study did not provide sufficient information to interpret the clinical relevance of these data.^51^

For interventions involving foot/ankle range of motion exercise components, three studies reported the changes in pain levels.^39^, ^41^, ^49^ One study reported improved (lower) pain scores in the intervention group compared to the control group by day 90, but did not supply raw data.^49^ Sallam et al.^39^ reported the mean pain score in the intervention group was 2.8/10 comparing with 3.6/10 in the control group by week 12. Davies et al.^41^ carried out a single‐group clinical trial in which the intervention group's median pain score decreased from 5.2/10 to 2/10 by week 24.

#### 3.6.8 Adverse events

For interventions involving resistance exercise components, five studies recorded adverse events during the trial period.^37^, ^38^, ^39^, ^42^, ^51^ Two studies reported no adverse events,^38^, ^39^ while two studies reported the discharge/exudate level from VLU increased after receiving PA interventions at first month and month 12.^38^, ^39^ One study found that 19 participants in the intervention group compared to three participants in the control group experienced one or more adverse events including ulcer deterioration, surrounding skin deterioration, development of new ulcer, pain, infection and hospitalisation.^42^

For interventions involving aerobic exercise/walking components, one study reported adverse events at month 12.^51^ In this study, two participants in the intervention group reported an increased discharge/exudate level from VLUs after receiving PA interventions.^51^

No adverse events were reported in the two studies that involved foot/ankle range of motion exercise components.^38^, ^39^

#### 3.6.9 Cost

Only one study reported on cost to health services and individuals at month 12.^51^ This study involved both resistance and aerobic/walking exercise components and found the total cost for the intervention group was £1931.76, compared to £3666.12 for the control group.^51^ The total cost to the healthcare service was £13825.60 for the intervention group, which is less than half of the cost for the control group (£48270.0).^51^

No data were reported for interventions involving foot/ankle range of motion exercise components.

### 3.7 Multicomponent interventions

Five studies described interventions involving more than one PA component (multicomponent) with their key findings summarised in Table 4.^39^, ^40^, ^41^, ^44^, ^51^ As shown in Table 4, there appeared a greater tendency for studies involving multicomponent interventions to yield clinically meaningful benefits compared to interventions involving single component. VLUs healing, measured by wound size, was greatly enhanced on day 12 and week 12 in participants who received interventions comprising resistance exercise and ankle/foot range of motion exercise.^39^, ^40^ The marked reduction in the pain score was also noticed in participants who received interventions comprising resistance exercise and ankle/foot range of motion exercise.^41^ Moderate improvements in VLU healing were observed in those who received multicomponent interventions involving resistance exercise and aerobic exercise/walking,^44^, ^51^ as well as all other outcomes in the limited number of studies that evaluated them.

**TABLE 4:** Synthesis of findings between interventions

Exercise types	Ulcer healing (e.g., time to healing, proportion of ulcer healed, rate of changes in ulcer areas)	Recurrence rate	Calf muscle pump function	Quality of life	Pain	Adverse events relate to exercise	Cost
Resistance exercise	√^1^ ?^3^		√^4^		?^1^	Reported^2^ N/R^1^	
Aerobic exercise/walking	?^1^						
Ankle/foot range of motion exercise	√^1^ ?^1^			√^1^ ?^1^	?^1^	N/R^1^	
Combination therapy (resistance exercise ± aerobic exercise walking ± ankle/foot range of motion exercise)	√^3^ ?^1^	?^1^		?^2^	√^2^ ?^1^	Reported^1^ N/R^1^	√^1^

*Note*: Superscript number denotes number of included studies reporting on that outcome; √ denotes likely better with PA treatment; ? denotes no apparent effect with treatment; N/R denotes no adverse event reported.

## 4 DISCUSSION

This scoping review is the first comprehensive summary of PA/exercise interventions applied as adjunct treatment for people with VLUs to offer a highly detailed evaluation of intervention components (in accordance with CERT recommendations) and explicitly distinguish observed treatment effects between clinically distinct PA/exercise components. Such detail has been lacking in previous reviews^18^, ^22^, ^23^ and may be a contributing factor to the challenges implementing earlier research into clinical practice. Our review identified a modest but emerging evidence base in this field (now 16 studies) based mostly upon interventions involving resistance training components. Findings suggest multicomponent interventions appear more effective than single‐component ones, particularly the combination of resistance training and foot/ankle range of motion exercises for improving VLU healing. The findings also reveal a lack of adequate reporting of PA interventions in this space in compliance with CERT. The absence of such specific information in academic literature may affect clinical uptake of PA/exercise interventions and undermine future research replication and is crucial to address in future research.

The predominance of resistance training interventions (11/16 studies) for this patient group is consistent with findings from earlier reviews.^18^, ^23^ The reason for this apparent preference is not entirely clear. Improvements in lower limb blood flow may be achieved following exercise training involving either walking/ aerobic training, resistance training or foot/ankle exercises due to their common involvement of the lower limbs and feet. Their mechanisms of action are, however, different. Aerobic training involving prolonged bouts of high repetition, low load activity focuses on improving endurance which may promote better cardiovascular health outcomes (e.g., microvascular epithelial changes, reduced peripheral resistance etc.).^36^, ^53^ Foot and ankle range of motion exercises aim to optimise ankle mobility and promote the calf muscle ‘pumping’ mechanism but are unlikely to confer many enduring effects once ceased.^21^ Resistance training involving shorter bouts of low repetition, higher load activity focuses on improving strength.^54^ This type of training may associate with sustained effects vascular re‐capillarisation, calf muscle hypertrophy and improved blood flow.^54^ Our review identified benefits on calf muscle pump function following resistance training but failed to identify studies involving other intervention types. It is important this ‘lack of evidence’ is not equated to ‘evidence of a lack of effect’.

The majority of interventions in our review involved home‐based, unsupervised PA components. Individuals with VLUs frequently required travel assistance, making home‐based PA interventions more viable for them.^55^ The results showed that studies with supervision had higher compliance rate and better clinical outcomes than those without supervision.^43^, ^50^, ^51^ As most study participants with VLUs had not previously received PA as an adjunct treatment, supervised interventions may be advisable in order to ensure appropriate individualisation of treatment dosage, appropriate monitoring of progressive overload and adequate support to facilitate effective behaviour change.^55^ As pointed out by previous authors that compliance is important to monitor in studies of PA interventions for fidelity checking, and to ensure any observed treatments can be potentially attributed to that treatment component in the case of PA being an adjunct component to other aspects of care (e.g., compression therapy).^22^ Surprisingly, no studies involving telemonitoring were included in this review despite the potential advantages that remotely supervised or supported therapy can offer (particularly during the more recent times of the COVID‐19 pandemic). A study by Kelechi et al.^56^ involving a small sample (*n* = 17) of people with healed VLUs and a short intervention duration (6 weeks) showed one‐on‐one tele coaching associated with a reported adherence rate of 100%. It remains unclear whether tele coaching would be acceptable to use for longer duration and whether it would confer clinically important effects on health outcomes in people with active VLUs.

This review also summarised the methods used to examine the key outcomes of interest in previous studies. The instruments and metrics used to measure pain and calf muscle pump function were highly consistent across different studies. In comparison, the instruments used to measure QoL were slightly different, and can be generally categorised into two types: generic health‐related instruments and disease‐specific instruments. Some studies reported diseases‐specific tools were more suitable and valid than generic instruments for VLU patients.^57^, ^58^ However, another study had the opposite opinion^59^ and reported that the ability of diseases‐specific tools to identify whether the changes in health‐related QoL caused by open ulcers and/or underlying causes were unclear. We did not identify any obvious differences between studies adopting generic instruments and diseases‐specific QoL instruments, and the QoL scores in intervention groups increased moderately after receiving PA/exercise interventions.^44^, ^46^, ^49^, ^51^ Overall, there was little variability in the instruments and metrics used to evaluate key outcomes of interest. It would certainly be beneficial to have clarity in the use of QoL instruments.^60^

Multicomponent interventions appear to confer benefits across many clinically important outcomes in the limited number of included studies (Table 4). The improvements in VLU healing (reductions in ulcer size as early as day 12^40^) were particularly noteworthy in participants who undertook resistance and foot/ankle range of motion exercise components. The effects of multicomponent interventions involving aerobic training were also favourable but less clear for the outcomes of ulcer healing.^18^ These findings are interesting, and contrast with apparently poorer outcomes following interventions comprising similar components in isolation. Optimising wound healing is a fundamental, time sensitive goal of treatment. Comprehensive PA treatment packages involving combinations of resistance, aerobic and foot/ankle range of motion training may help accelerate this process more than individual components due to their ability to target the same goal via a multifaceted approach. This can be important for people affected by VLUs who often report difficulty adhering to compression and other therapies. Additional research appears indicated to confirm the merit of multicomponent PA interventions.

This review identified some key gaps in knowledge worthy of future attention. While most studies reported the effect of PA/exercise interventions on ulcer healing status, the impact on ulcer recurrence has rarely been investigated. Only one study measured ulcer recurrence rates 12 months following completion of an intervention involving resistance and aerobic exercise/walking training components; however, no clinical difference was detected (intervention group: 12% vs. control group: 5%).^46^ The lack of evidence relating to VLU recurrence has been previously identified^18^, ^22^, ^23^ and remains a topic of unmet need. Uncertainty also remains regarding the precise role of PA interventions on longer term health outcomes. While a lack of long‐term follow‐up in studies of people with VLUs has been previously described,^22^ we know very little of the relative contribution of PA to important outcomes relating to healing and recurrence, including their potential enduring effects following treatment cessation. Resistance exercise has been proposed as potentially having such a role due to its potential impact on calf muscle pump function,^61^ and previous studies have associated limited ankle range of motion with increased risk of future ulcer recurrence.^62^, ^63^ Confirmation of a causative effect would appear to require incorporation of serial measures of ‘mechanistic’ outcomes such as PA levels, muscle strength or calf‐muscle pump function in conjunction with follow‐up clinical outcomes over the total follow‐up period (until final outcome measurement). Issues such as age, frailty, mobility, BMI may affect one's ability to undertake PA and could explain some heterogeneity of findings among studies. No studies tailored interventions according to these factors; however, this would be a valuable area of research to explore in future studies.

### 4.1 Future research and implications

Our findings suggest interventions involving a combination of training components may confer greater benefit than single elements alone. The effectiveness of such an approach, however, requires further attention integrating perspectives of multiple stakeholders and sufficient sample sizes to ensure adequate statistical power for key outcomes such as those used within this review. Future research should also examine the long‐term impact of this combination of PA therapies on calf muscle pump function. Furthermore, it is strongly advised that future clinical trials adhere to a well‐structured template (i.e., CERT) when reporting the characteristics of PA/exercise therapies. The results of this review identify a gap existing in the web‐based approach to deliver exercise programmes. Given that this patient population frequently require assistance when travelling, and that researchers face existing challenges such as COVID‐19 when doing clinical trials, future clinical studies could explore the feasibility and acceptability of incorporating web‐based elements into PA programmes. A systematic review focusing on qualitative evidence is also necessary to investigate the barriers and enablers to PA involvement in patients with VLUs,^64^ and the findings of this review will aid in the design of feasible and acceptable PA interventions for future clinical trials.

### 4.2 Strengths and Limitations

Key strengths of this review included the incorporation of a variety of study designs and strong emphasis on critical components of PA/exercise interventions (e.g., CERT). Furthermore, the choice of review outcomes provides an important framework that should guide future studies involving PA interventions for people with VLUs.

There are some limitations in this review. Even though we conducted an extensive literature search using a wide range of electronic databases combined with hand‐searching of grey literature sources, it is possible we may have missed some studies. We encourage readers or other researchers who may have reported in this space to contact us. Further, we had little opportunity to consult with external healthcare professionals as a result of the unanticipated pandemic. We also omitted PA levels as an outcome of this review in our protocol despite its obvious relevance; however, no studies reported such data. It remains clinically valuable to understand whether PA interventions influence PA behaviours in people affected by VLU; however, the focus of this review was the potential for PA interventions to affect outcomes primarily related to VLU healing rather than PA levels.

## 5 CONCLUSIONS

Resistance exercise is the most commonly examined PA/exercise intervention component for people with VLUs; however, the evidence base is still young (but maturing). Interventions involving resistance exercise and foot/ankle range of motion exercise components may be a clinically effective adjunct treatment for improving ulcer size and calf muscle pump function; however, the effects on ulcer recurrence remain unknown. More research is needed to confirm the most appropriate model of care involving PA interventions to optimise outcomes for people with VLU the feasibility and acceptability of this combined PA/exercise regimen, as well as its effects on ulcer healing and recurrence.

## CONFLICT OF INTEREST

The authors declare that they have no conflict of interest.

## AUTHOR CONTRIBUTIONS

Yunjing Qiu and Carolina D. Weller created the project and the key conceptual ideas. All authors contributed to the design, and the frameworks for this review. Yunjing Qiu and Victoria Team screened the articles for eligibility and extracted the relevant data from eligible studies. Yunjing Qiu wrote the manuscript with assistance from Christian R. Osadnik, Victoria Team and Carolina D. Weller. All authors provided critical feedback, and reviewed and approved the final version of the manuscript.

## Supporting information


**Appendix** **S1.** Supporting Information

## Data Availability

This scoping review uses data from publically available sources.
