# Supplementary material for: Effects of physical activity as an adjunct treatment on healing outcomes and recurrence of venous leg ulcers: A scoping review
Source: Wound Repair Regen. 2022 Feb 10;30(2):172–85. doi: 10.1111/wrr.12995 (PMC9303258; doi:10.1111/wrr.12995)
Supplement: Supplementary file 1 — Appendix S1. Supporting Information [file WRR-30-172-s001.pdf]

Additional File 1: PRISMA-ScR checklist

Preferred Reporting Items for Systematic reviews and Meta-Analyses extension for Scoping Reviews (PRISMA-ScR) Checklist

SECTION	ITEM	PRISMA-ScR CHECKLIST ITEM	REPORTED ON PAGE #
**TITLE**			
Title	1	Identify the report as a scoping review.	1
**ABSTRACT**			
Structured summary	2	Provide a structured summary that includes (as applicable): background, objectives, eligibility criteria, sources of evidence, charting methods, results, and conclusions that relate to the review questions and objectives.	1-2
**INTRODUCTION**			
Rationale	3	Describe the rationale for the review in the context of what is already known. Explain why the review questions/objectives lend themselves to a scoping review approach.	3-4
Objectives	4	Provide an explicit statement of the questions and objectives being addressed with reference to their key elements (e.g., population or participants, concepts, and context) or other relevant key elements used to conceptualize the review questions and/or objectives.	4
**METHODS**			
Protocol and registration	5	Indicate whether a review protocol exists; state if and where it can be accessed (e.g., a Web address); and if available, provide registration information, including the registration number.	4
Eligibility criteria	6	Specify characteristics of the sources of evidence used as eligibility criteria (e.g., years considered, language, and publication status), and provide a rationale.	Table 1
Information sources*	7	Describe all information sources in the search (e.g., databases with dates of coverage and contact with authors to identify additional sources), as well as the date the most recent search was executed.	5-6
Search	8	Present the full electronic search strategy for at least 1 database, including any limits used, such that it could be repeated.	Additional File 2
Selection of sources of evidence†	9	State the process for selecting sources of evidence (i.e., screening and eligibility) included in the scoping review.	8

SECTION	ITEM	PRISMA-ScR CHECKLIST ITEM	REPORTED ON PAGE #
Data charting process‡	10	Describe the methods of charting data from the included sources of evidence (e.g., calibrated forms or forms that have been tested by the team before their use, and whether data charting was done independently or in duplicate) and any processes for obtaining and confirming data from investigators.	8
Data items	11	List and define all variables for which data were sought and any assumptions and simplifications made.	8
Critical appraisal of individual sources of evidence§	12	If done, provide a rationale for conducting a critical appraisal of included sources of evidence; describe the methods used and how this information was used in any data synthesis (if appropriate).	Not applicable
Synthesis of results	13	Describe the methods of handling and summarizing the data that were charted.	9-10
**RESULTS**			
Selection of sources of evidence	14	Give numbers of sources of evidence screened, assessed for eligibility, and included in the review, with reasons for exclusions at each stage, ideally using a flow diagram.	10-11<br>Fig. 1
Characteristics of sources of evidence	15	For each source of evidence, present characteristics for which data were charted and provide the citations.	11<br>Additional File 3,4
Critical appraisal within sources of evidence	16	If done, present data on critical appraisal of included sources of evidence (see item 12).	Not applicable
Results of individual sources of evidence	17	For each included source of evidence, present the relevant data that were charted that relate to the review questions and objectives.	13-19<br>Table 3<br>Additional File 3,5
Synthesis of results	18	Summarize and/or present the charting results as they relate to the review questions and objectives.	19-20<br>Table 4<br>Additional File 6
**DISCUSSION**			

SECTION	ITEM	PRISMA-ScR CHECKLIST ITEM	REPORTED ON PAGE #
Summary of evidence	19	Summarize the main results (including an overview of concepts, themes, and types of evidence available), link to the review questions and objectives, and consider the relevance to key groups.	22-27
Limitations	20	Discuss the limitations of the scoping review process.	27
Conclusions	21	Provide a general interpretation of the results with respect to the review questions and objectives, as well as potential implications and/or next steps.	27-28
**FUNDING**			
Funding	22	Describe sources of funding for the included sources of evidence, as well as sources of funding for the scoping review. Describe the role of the funders of the scoping review.	28

JBI = Joanna Briggs Institute; PRISMA-ScR = Preferred Reporting Items for Systematic reviews and Meta-Analyses extension for Scoping Reviews.

* Where *sources of evidence* (see second footnote) are compiled from, such as bibliographic databases, social media platforms, and Web sites.

† A more inclusive/heterogeneous term used to account for the different types of evidence or data sources (e.g., quantitative and/or qualitative research, expert opinion, and policy documents) that may be eligible in a scoping review as opposed to only studies. This is not to be confused with *information sources* (see first footnote).

‡ The frameworks by Arksey and O'Malley (6) and Levac and colleagues (7) and the JBI guidance (4, 5) refer to the process of data extraction in a scoping review as data charting.

§ The process of systematically examining research evidence to assess its validity, results, and relevance before using it to inform a decision. This term is used for items 12 and 19 instead of "risk of bias" (which is more applicable to systematic reviews of interventions) to include and acknowledge the various sources of evidence that may be used in a scoping review (e.g., quantitative and/or qualitative research, expert opinion, and policy document).

From: Tricco AC, Lillie E, Zarin W, O'Brien KK, Colquhoun H, Levac D, et al. PRISMA Extension for Scoping Reviews (PRISMAScR): Checklist and Explanation. Ann Intern Med. 2018;169:467–473. doi: 10.7326/M18-0850.

Additional File 2: Search strategy

Supplementary Table 1: Searching strategy for Ovid MEDLINE

#	Searches
1	leg ulcer/ or varicose ulcer/
2	(varicose or venous or leg or stasis or crural or lower extremit* orlow limb) adj2 ulcer*
3	Ulc* cruris
4	1 or 2 or 3
5	Rehabilitation/or Cardiac Rehabilitation/
6	"Activities of Daily Living"/
7	Early Ambulation/
8	Recreation therapy/ or telerehabilitation/ or physical therapy modalities/
9	tele rehabilitation* or telerehabilitation* or remote rehabilitation* or virtual rehabilitation*
10	exp exercise/
11	exp exercise therapy/
12	exp exercise movement techniques/

13	(strength* or recreation* or rehabilitat* or endurance or physical or resistan* or weight bearing or weight resistan* or isometric*) adj3 (therap* or program* or training* or exercis* or activit*)
14	walk* or run* or jog* or aerobic* or pilates or tai chi or yoga
15	calf rais* or heel rais* or calf muscle pump*
16	5 or 6 or 7 or 8 or 9 or 10 or 11 or 12 or 13 or 14 or 15
17	4 and 16
18	limit 17 to English language

Supplementary Table 2: Searching strategy for CINAHL Plus

#	**Searches**
S15	S4 AND 13 Narrow by Language: - english
S14	S4 AND S13
S13	S5 OR S6 OR S7 OR S8 OR S9 OR S10 OR S11 OR S12
S12	(strength* or recreation* or rehabilitat* or endurance or physical or resistan* or weight bearing or weight resistan* or isometric*) N3 (therap* or program* or training* or exercis* or activit*)
S11	calf rais* or heel rais* or calf muscle pump*
S10	walk* or run* or jog* or aerobic* or pilates or tai chi or yoga

S9	(MH "Exercise") OR (MH "Therapeutic Exercise")
S8	TX tele rehabilitation* or telerehabilitation* or remote rehabilitation* or virtual rehabilitation*
S7	(MH "Recreational Therapy") OR (MH "Telerehabilitation") OR (MH "Physical Therapy")
S6	(MH "Rehabilitation, Cardiac") OR (MH "Activities of Daily Living") OR (MH "Early Ambulation")
S5	(MH "Rehabilitation")
S4	S1 OR S2 OR S3
S3	TX Ulc* cruris
S2	TX varicose ulcer* OR venous ulcer*OR leg ulcer* OR stasis ulcer* OR lower extremit* ulcer* OR low limb ulcer*
S1	(MH "Venous Ulcer") OR (MH "Leg Ulcer")

Supplementary Table3: Searching strategy for Cochrane Central Library

**#**	**Searches**
1	MeSH descriptor: [Varicose Ulcer] explode all trees
2	MeSH descriptor: [Leg Ulcer] explode all trees

3	(varicose or venous or leg or stasis or crural or lower extremit* orlow limb) adj2 ulcer*
4	#1 OR #2 OR #3
5	MeSH descriptor: [Rehabilitation] explode all trees
6	MeSH descriptor: [Cardiac Rehabilitation] explode all trees
7	MeSH descriptor: [Activities of Daily Living] explode all trees
8	MeSH descriptor: [Early Ambulation] explode all trees
9	MeSH descriptor: [Recreation Therapy] explode all trees
10	MeSH descriptor: [Telerehabilitation] explode all trees
11	MeSH descriptor: [Physical Therapy Modalities] explode all trees
12	tele rehabilitation* or telerehabilitation* or remote rehabilitation* or virtual rehabilitation*
13	MeSH descriptor: [Exercise] explode all trees
14	MeSH descriptor: [Exercise Therapy] explode all trees
15	MeSH descriptor: [Exercise Movement Techniques] explode all trees
16	(strength* or recreation* or rehabilitat* or endurance or physical or resistan* or weight bearing or weight resistan* or isometric*) adj3 (therap* or program* or training* or exercis* or activit*)
17	walk* or run* or jog* or aerobic* or pilates or tai chi or yoga

18	calf rais* or heel rais* or calf muscle pump*
19	#5 OR #6 OR #7 OR #8 OR #9 OR #10 OR #11 OR #12 OR #13 OR #14 OR #15 OR #16 OR #17 OR #18
20	#4 AND #19

Additional File 3: *characteristics of included studies*

Title	Author (y)	Country	Study Design	Study duration	Study group	Number of recruited participants	Number of analysed participants	Population	Intervention types	Metrics &instruments used to evaluate outcomes of interest	Funding	Notes
Effect of exercise on calf muscle pump function in patients with chronic venous disease	Yang (1999) [47]	Australia	Single-armed pilot study	6 wks	Single group	IG: 20	IG: 20	Patients with chronic VLUs.	Tip-toe exercise in addition to compression.<br><br>Compression type: N/R.	Calf muscle pump function: ejection venous volume (mls); EF (percentage); residual venous volume (mls); RVF (percentage).<br><br>APG is used for measurement.	N/R	No compression bandages or stocking during exercise.
Hemodynamic Effects of Supervised Calf Muscle Exercise in Patients with Venous Leg Ulceration	Kan (2001) [43]	United Kingdom	Prospective controlled study	7 ds	Parallel group	21<br>IG:10<br>CG: 11	21<br>IG:10<br>CG: 11	Patients with VLUs.	IG: Calf muscle exercise in addition to compression.<br><br>CG: compression<br><br>Compression type: short stretched compression bandage.	Calf muscle pump function: ejection venous volume (mls); EF (percentage); residual venous volume (mls); RVF (percentage).<br><br>APG-1000 is used for measurement.	N/R	N/A

A home-based exercise programme improves ankle range of motion in long-term venous ulcer patients	Davies (2007) [41]	London	Single-armed pilot study	24 wks	Single group	IG: 11	IG: 10	Patients with VLUs.	IG: 5-10 mins exercise program in addition to compression bandaging<br><br>Compression type: N/R	Pain: a sample scale of 1-10.	N/R	The Hygenic Corporation (Akron, Ohio, USA) for donating the Thera Bandst and handles.<br><br>Bandaging off when conducting exercises.<br>1-2 sessions per week with bandaging on when exercising.
The PREPARE pilot RCT of homebased progressive resistance exercises for venous leg ulcers	Jull (2009) [42]	New Zealand	RCT (open label)	12 wks	Parallel group	40<br>IG: 21<br>CG:19	39<br>IG: 20<br>CG:19	Adults ≥ 18 years old a clinically diagnosed VLU.	IG: Progressive resistance exercise in addition to compression.<br><br>CG: compression<br><br>Compression type: individual tailored	Calf muscle pump function: APG<br><br>Ulcer status: Silhouette Mobile device (ARANZ)	The Health Research Council of New Zealand	N/A
An experimental study of prescribed walking in the management of venous leg ulcers	Meagher (2012) [48]	Ireland	RCT	12 wks	Parallel group	40<br>IG:18<br>CG: 17	35<br>IG: 18<br>CG:17	Adults ≥ 18 years old a clinically diagnosed VLU.	IG: 10 000 steps per day in addition to compression<br><br>CG: compression<br><br>Compression type: multilayer compression bandaging	Pain: VAS<br><br>Ulcer status: VISITRAK digital planimetry.	No external sources of funding	N/A

Anodyne therapy versus exercise therapy in improving the healing rates of venous leg ulcer	Ahmed (2013) [40]	Egypt	RCT	12ds	Four equal groups.	40<br>IG:10<br>CG: 10	40<br>IG:10<br>CG: 10	Patients with a clinically diagnosed VLU	IG: resisted exercise in addition to conventional VLU treatment<br><br>CG: conventional VLU treatment<br><br>Compression type: high compression bandaging	Ulcer status: PUSH	No external sources of funding	Conventional VLU treatment: cleaning ulcer with normal saline and compression
A home-based progressive resistance exercise programme for patients with venous leg ulcers: a feasibility study	O'Brien (2013) [38]	Australia	RCT (open-label)	12 wks	Parallel group	13<br>IG: 6<br>CG: 7	11<br>IG: 6<br>CG: 5	Adults ≥ 18 years old a clinically diagnosed VLU.	IG: home-based progressive resistance exercise in addition to compression<br><br>CG: compression<br><br>Compression type: multilayer compression bandaging	Rate of changes in the ulcer size: wound tracings and a portable digital planimetry device.<br><br>Calf muscle pump function: APG-1000.	N/R	This study and a National Health and Medical Research Council (NHMRC) funded study share all eligible patients.
Value of combined exercise and ultrasound as an adjunct to compression therapy in chronic venous leg ulcers	Sallam (2017) [39]	Egypt	RCT	12 wks	Four equal groups	60<br>IG: 15<br>CG:15	60<br>IG: 15<br>CG:15	Patients with a clinically diagnosed VLU	IG: exercise in addition to compression<br><br>CG: compression<br><br>Compression type: elastic multilayer dressing	Ulcer size: multiplying the maximum width and length of the ulcer	No external sources of funding	N/A
Evaluating the effectiveness of a self-	O'Brien (2017) [44]	Australia	RCT (open-label)	12 wks	Parallel group	63<br>CG: 32<br>IG: 31	59<br>CG: 30<br>IG:29	Patients with a clinical	IG: progressive resisted exercise,	Proportion of ulcer healed: wound tracings and a	N/R	N/A

management exercise intervention on wound healing, functional ability and health-related quality of life outcomes in adults with venous leg ulcers: a randomised controlled trial								diagnosed VLU.	walking in addition to compression<br><br>CG: compression<br><br>Compression type: multilayer compression bandaging	portable digital planimetry device (VISITRAK digital)<br><br>QoL: SF-8 questionnaires		
Effectiveness of the strategies of an orientation programme for the lifestyle and wound-healing process in patients with venous ulcer: A randomised controlled trial	Domingues (2018) [49]	Brazil	RCT (single-blind)	12 wks	Parallel group	102<br>IG:49<br>CG:53	71<br>IG:35<br>CG:36	Patients with a clinical diagnosed VLU.	IG: exercise for lower extremities in addition to compression<br><br>CG: compression<br><br>Compression type: N/R	QoL: The abbreviated version of the FLQAw.<br><br>Pain: numeric pain rating scale.<br><br>Ulcer status: PUSH score.	Fundacão de Amparo a Pesquisa do Estado de Sao Paulo-FAPESP (2015/15134-8).	N/A
Supervised exercise training as an adjunct therapy for	Klonizakis (2018) [51]	United Kingdom	RCT	12wks	Parallel group	39<br>IG:18<br>CG:21	39<br>IG:18<br>CG:21 (at 3 mths)	Adults ≥ 18 years old a clinically diagnosed VLU.	IG: Exercise in addition to compression.<br><br>CG: compression	QoL: EQ-5D-5L and VEINES-QOL.	The National Institute for Health Research	N/A

venous leg ulcers: a randomized controlled feasibility trial							CG:20 (at 6 and 12 mths)		Compression type: N/S			
The influence of exercise on ulcer healing in patients with chronic venous insufficiency	Mutlak (2018) [45]	United Kingdom	RCT	12 wks	Four equal groups	80<br>CG:20<br>IG 20	80<br>CG:20<br>IG 20	Patients with a clinical diagnosed VLU.	IG: ankle mobility exercise in addition to compression.<br>CG: compression<br><br>Compression type: N/R	Rate of changes in the ulcer size: a cleaned metal ruler was used to measure the widest and longest dimensions of ulcer areas.	The Vascular Department of Hammersmith Hospital	N/A
Effect of Ankle Resisted Exercises on Venous Leg Ulcers Healing	Nabil (2019) [50]	Egypt	RCT	12 wks	Parallel group	30<br>IG: 15<br>CG: 15	30<br>IG: 15<br>CG: 15	Adult aged between 45-60 years with 2nd and 3rd degree chronic VLUs.	IG: ankle resistance exercise in addition to traditional medical treatment<br><br>CG: traditional medical treatment<br><br>Compression type: N/R	Rate of changes in the ulcer size: Injecting sterilized saline into ulcers and measure the volume by cubic centimetre.	N/R	Traditional medical treatment: compression, ultrasound application, medical treatment and ROM ex.
A multi-centre, prospective, randomised controlled feasibility study of plantar resistance exercise therapy for venous leg ulcers –	Jonker (2020) [37]	England	RCT	12 wks	Parallel group	32<br>IG:15<br>CG:17	31<br>IG:15<br>CG:16	Adults ≥ 18 years old a clinically diagnosed VLU.	IG: Seated plantar resistance exercise in addition to compression<br><br>CG: compression<br><br>Compression type: N/R	Ulcer healing status: using transparent wound measurement sheet; Acrobat reader was used for calculation of ulcer areas.<br><br>Wound size: PUSH score.<br><br>Pain: VAS	No external funding for this study.	A research grant was received from Steplt System AB, Sweden for providing Step-It pedals only.

Results of the PREVUE study										Quality of life: the Charing Cross Venous Ulcer Questionnaire		
FOOTFIT Physical Activity mHealth Intervention for Minimally Ambulatory Individuals with Venous Leg Ulcers: A Randomized	Kelechi (2020) [46]	United States	RCT	6 wks	Parallel group	24 Footfit:12 Footfit +app: 12	23 Footfit: 11; Footfit +app 12.	Adult ≥ 18 years old with a clinically diagnosed VLU.	FOOTFIT: progressive exercise in addition to compression<br><br>FOOTFIT+ app: progressive exercise in addition to compression; app has communication feature.	QoL: SF-12 questionnaires	The National Institutes of Health and National Center for Advancing Translational Sciences of the National Institutes of Health	Both groups received the exercise program.
Ongoing study												
Impact of Physical Activity as a Coadjuvant Treatment in the Healing of Venous Ulcers in Primary Health Care: Multicenter Randomized Clinical Trial - Active Legs Project	Herraiz - Ahijado Álvarez (02/ 2021) [52]	Spain	RCT (open label)	6 mths	Parallel group	224 IG: 112 CG: 112	N/A	Patients with VLUs	IG: lower limb exercise, walking, usual care and compression.<br><br>CG: usual care and compression<br><br>Compression type: multilayer compression	Changes in ulcer area: using digital photography for measurement; using the Vistrack device for calculation.<br><br>QoL: the Charing Cross Venous Ulcer Questionnaire<br><br>Pain: visual analogical scale of the McGill questionnaire	N/R	Usual care: assessment, cleaning, debridement, topical treatment.

Note: air plethysmography, APG; control group, CG; day(s), d(s); ejection fraction, EF; five-level EuroQol five-dimensional questionnaire, EQ-5D-5L; intervention group, IG; minutes, mins; millilitres, mls; months, mths; wks, weeks; not reported, N/R; not applicable, N/A; pressure ulcer scale for healing, PUSH; randomized control trial, RCT; residual volume fraction, RVF; short form-8, SF-8; short form-12, SF-12; visual analogue pain scale, VAS; venous leg ulcer(s), VLU(s); venous insufficiency epidemiological and economic study on quality of life, VEINES-QoL.

Additional File 4: *Baseline participant characteristics in the included studies*

Author (y)	Age (y)	Gender (M/F)	BMI (mean) (kg/m²)	No. of present ulcers	Ulcer area (cm²)	Ulcer duration	Mobility
Yang (1999) [47]	Median (range)<br>68 (34-88)	IG: 6/36<br>CG:16/ 44	N/R	N/R	N/R	N/R	N/R
Kan (2001) [43]	Median (range)<br><br>IG: 72 (49-81)<br>CG: 80 (69-85)<br>p>0.09	N/R	N/R	N/R	Median<br>IG:16.5 (4-50)<br>CG: 20 (10-33)	Median (mth)<br>IG: 4 (13-52)<br>CG: 29 (19-41)	N/R
Davies (2007) [41]	Median (range)<br>73(63-80).	IG: 4/6	N/R	N/R	Median<br>IG: 9.8 (1.5-93)	Median (y)<br>IG: 20 (6-51)	
Jull (2009) [42]	Mean (SD)<br>IG: 54.6 (19.9)<br>CG: 53.3 (19.9)	IG:5/16<br>CG:8/11	N/R	N/R	Median<br>IG: 3.4 (1.4–6.0)<br>CG: 3.1 (1.1–4.8)	Median (wk)<br>IG:23.0 (8–68)<br>CG: 28.0 (10–64)	Mobilise freely without aids<br>IG:18 (85.7%)<br>CG:16(84.2%)
Meagher (2012) [48]	Median (range)<br>IG: 66 (32–84)<br>CG: 78 (55–91)<br>p=0.002.	IG:<br>6(33%)/12(67%)<br>CG:<br>4(24%)/13(67%).	N/R	1 ulcer:<br>IG :12 (67%)<br>CG:10 (59%)<br><br>1–5 ulcers:<br>IG: 6 (33%)<br>CG: 7 (41%)	< 10cm2<br>IG:17 (94%)<br>CG:11 (65%)<br><br>> 10cm<br>IG: 1 (5.6%)<br>CG:26 (35%)	Median (wk)<br>IG: 8.5<br>CG: 15	Fully independently mobile:<br>CG:14 (82%)<br>IG17 (94%)<br><br>Minimal assistance with one stick:<br>CG: 3(18%)<br>IG:1(5.6%)
Ahmed (2013) [40]	Mean ±SD<br>IG: 58.70±4.11<br>CG:60.00±4.94	IG: 5/5<br>CG: 6/4	N/R	N/R	N/R	Mean (mth)<br>IG:5.70± 2.35<br>CG: 6.70±1.41.	N/R
O'Brien (2013) [38]	Mean (SD)<br>IG:66(6)<br>CG: 63.6(20)	IG: 3/3<br>CG: 3/2	IG: 31·7(6·6) CG: 36·7 (12·3)	N/R	Mean<br>IG:5.1 (5.5)<br>CG:3.2 (3.9)	Mean (wk)<br>IG: 19·5 (14)<br>CG: 34·8 (17)	Mobility (with aid):<br>IG:2<br>CG:2
Sallam (2017) [39]	Mean ±SD<br>IG: 51.2± 6.5<br>CG: 51.3±6.8	IG:7/8<br>CG: 6/9	IG:30.5±4.2;<br>CG:30.1±4.5	N/R	Mean<br>IG:12.9±5.7<br>CG:14.1±5.2	Mean (mth)<br>IG:15.5±1.8<br>CG:15.1±1.9	N/R

O'Brien (2017) [44]	Mean ±SD<br>IG:71.3 ±15·8<br>CG: 71·7±13·4	IG: 32(51.6%)/ 30(48.4%).<br>CG: NR.	N/R	N/R	Mean<br>IG: 8·8 (±1·4)<br>CG: 6·0 (±8·3)	Median (wk)<br>CG:14 (3–234) IG:16 (2–416)	N/R
Domingues (2018) [49]	Mean (SD)<br>CG: 68.17 (12.63)<br>IG: 64.83 (12.86)	Female<br>IG: 21 (60.00%);<br>CG:20 (55.56%).	N/R	N/R	N/R	Mean (mth)<br>CG: 55.89 (67.62)<br>IG: 58.57 (81.47)	N/R
Klonizakis (2018) [51]	Mean ± SD<br>IG: 65.4 ±14.9;<br>CG:61.9 ±10.9	IG:9/9<br>CG:14/7	N/R	N/R	Median<br>IG: 4.9 (1.9–136.4)<br>CG: 5.7 (1.3–56.6)	Mean (mth)<br>IG:12.7 ± 19.9 CG:7.1 ± 8.1	Walking with difficulty:<br>IG:8 (44); CG:10 (48).<br><br>No walking: IG:5 (28); CG: 7 (33).
Mutlak (2018) [45]	Mean ±SD<br>CG:69.15±12.05<br>IG: 62.15±14.05	IG:10/10<br>CG:10/10	CG:32.40±3.84<br>IG: 30.85±4.28	N/R	Median<br>CG: 2.52 (4.18-0.63)<br>IG: 2.39 (3.51-1.16)	N/R	N/R
Nabil (2019) [50]	Mean<br>IG: 52.8;<br>CG: 51.33	IG: 11/4<br>CG: 9/6	N/R		Mean<br>IG: 2.88 ± 0.83 cm^3<br>CG:2.96±0.72 cm^3	N/R	N/R
Jonker (2020) [37]	Median (range)<br>IG:73 (69-78)<br>CG:77 (71–82)	IG: 7/8<br>CG:5/12	CG: 30.1<br>IG: 30.6		Median:<br>IG: 3.76<br>CG: 4.39	Mean (wk)<br>IG: 42<br>CG: 20	N/R
Kelechi (2020) [46]	Mean ±SD<br>FOOTFIT: 60.7± 13.7;<br>FOOTFIT+ app: 69.1 ± 11.5	FOOTFIT: 66.7% (8/12);<br>FOOTFIT+: 50.0% (6/12).	FOOTFIT: 45.2 ± 14.5<br><br>FOOTFIT+ app: 35.5 ± 8.8	Mean<br>FOOTFIT: 7.2 ± 4.9<br><br>FOOTFIT+ app: 5.2 ± 9.8	N/R	Mean (mth)<br>FOOTFIT: 35.0 ± 36.7<br><br>FOOTFIT+ app: 27.1 ± 31.0	N/R

Note: body mass index, BMI; control group, CG; female, F; intervention group, IG; male, M; month, mth; not reported, NR; standard deviation, SD; week, wk; year, y.

Additional File 5: *Characteristics of PA/ exercise interventions used in included studies following CERT*

Author (y)	Exercise types	Exercise setting	Supervised / Unsupervised<br><br>Supervisors	Individually /Group	Exercise equipment	Generic/ Individual tailored	visual materials on exercise technique	Starting level of exercise	Decision rules for progressing the exercise program	Motivation strategies	Measurement of adherence/concordance/retention/attendance /participation/completion	Adherence concordance/retention /attendance rate/ participation	Program modification
Yang (1999) [47]	Foot/ankle ROM	Home	Unsupervised	Individually	N/R	Individual tailor	N/R	Half number of the maximum number of tip-toe exercises that participants could conduct initially.	N/R	N/R	N/R	N/R	N/R
Kan (2001) [43]	RT with progressive overload	University-associated tertiary care hospital.	Supervised (Supervisors: N/R)	N/R	a 4kgs resistance pedal ergometer	Generic	N/R	First three days: ¾ of the maximal number of flexions reached at the baselines for six mins at the rate of 1 flexion per second.	N/R	N/R	N/R	N/R	N/R

Davies (2007) [41]	RT with progressive overload; Foot/ankle ROM	Home	Unsupervised	Individually	Elastic resistance bands	Generic	N/R	Participants started on resistance band based on their ability, and then gradually increased to the next colour band.	N/R	N/R	Exercise diaries	IG: 7 had excellent compliance to the exercise.	No modification.
Jull (2009) [42]	RT with progressive overload	Home	Unsupervised	Individually	No additional equipment is needed	Individual tailored	N/R	Research nurse assessed each individual's level, then prescribed regimens at participants' maximum.	Research nurses assessed the maximal number of the heel raises of each participant at baseline, 3, 6 and 9 weeks and prescribed a regimen at 80% of the participant's maximum.	N/R	Exercise diaries	IG: 81%	N/R

Meagher (2012) [48]	Aerobic training	Home	Unsupervised	Individually	N/R	Generic	N/R	N/R	N/R	N/R	Digi walker pedometer for self-reporting; ActivPal monitor at week4 for continuing 7 days	IG: 6 (33%) reach the target.	N/R
Ahmed (2013) [40]	RT; Foot/ankle ROM	A clinic at Naser Hospital, Cairo, Egypt	N/R	N/R	N/R	Generic	N/R	N/R	N/R	N/R	N/R	N/R	N/R
O'Brien (2013) [38]	RT with progressive overload	Home	Unsupervised	Individually	No additional equipment was needed.	Individually tailored	No visual material was provided. (Exercise regime is prescribed face-to-face / over the phone.	All participants started at stage 1.	Participants moved on to the next level when they have successfully completed the current level for at least 3 ds or encouraged by the author.	N/R	N/R	N/R	N/R

Sallam (2017) [39]	RT with progressive overload; foot and ankle ROM	N/R	N/R	N/R	Elastic resistance bands	Generic	N/R	N/R	N/R	N/R	N/R	N/R	N/R
O'Brien (2017) [44]	RT with progressive overload; aerobic training	Home	Unsupervised	Individually	No additional equipment was needed.	Generic	Exercise booklets were provided to the IG.	See O'Brien (2013)	See O'Brien (2013)	Pedometer was used as a motivational tool for the IG.	Self-report	N/R	N/R
Domingues (2018) [49]	Foot/ankle ROM	Home	Unsupervised	Individually	N/R	Generic	Nursing guidelines were provided to the IG.	N/R	N/R	IG: 4 in-person meetings every 4 wks; phone calls b/w in-person meetings.	N/R	IG: 19 (59%) adhered to the exercise program.	N/R

Kloniz akis (2018) [51]	RT with progressive overload; aerobic training	Primary location: The Centre for Sport and Exercise Science at Sheffield Hallam University<br><br>Secondary location: Human Performance Centre at the University of Lincoln	Supervised<br><br>(exercise physiologists)	Group (4 participants/ session)	motorized treadmills; upright exercise bikes; dumbbells ranged 2-20 kg; height-adjustable step; medium-sized stability ball; seated leg press machine; Thera band.	Individual tailored	N/R	The exercise physiologist determined appropriate exercises for each participant according to the level of physical fitness and mobility.	Once the targeted frequency and duration was achieved, the intensity of specific exercise was progressed on an individual basis.<br><br>Aerobic exercises: aiming for an exertion level of 12-14.<br><br>Resistance exercises: aiming at moderate muscle fatigue within 10-15 repetitions.	N/R	Exercise session case report form	IG: 13(72%) completed all sessions.	No modification.
Mutlak (2018) [45]	Ankle/foot ROM training	Home	unsupervised	Individually	N/R	Generic	Leaflets were provided to the IG.	N/R	N/R	Diaries, leaflet, phone calls fortnightly	N/R	Overall adherence rate was 84%	N/R

Nabil, (2019) [50]	RT with progressive overload	N/R	Supervised (A trained exercise physiologist)	Group (4 participants/session)	Elastic bands, ankle cuffs, dumb-bells, free weight cuffs	Generic	N/R	N/R	N/R	N/R	N/R	N/R	No modification.
Jonker (2020) [37]	RT	home	Unsupervised	Individually	A 6kgs resistance steplt rocker pedal	N/R	N/R	N/R	N/R	Text messages were sent to participants every 2 wks.	N/R	N/R	N/R
Kelechi (2020) [46]	Ankle/foot ROM training	home	Unsupervised	Individually	N/R	Generic	N/R	N/R	N/R	Educational/ motivational messages were sent to both groups.	N/R	N/R	No modification.
Herraiz-Ahijado (2021) [52]	Aerobic training	Home	unsupervised	Individually	N/R	N/R	N/R	N/R	N/R	N/R	Self-reported activity diaries.	N/R	N/R

Notes: between, b/w; day(s), d(s); intervention group, IG; not reported, N/R; range of motion, ROM; resistance training, RT; week(s), wk(s).

Additional File 6: *Summary of study results*

	Primary outcomes				Secondary outcomes					
**Author (y)**	**Time to healing (wk)**	**Proportion of ulcer healed [N (%)]**	**Rate of changes in the area of the ulcer**	**Recurrence rate [N (%)]**	**Calf muscle pump function [EF (%); RVF (%)]**	**Quality of life**	**Pain**	**Adverse events relate to exercise**	**Individual cost**	**Health service cost**
Yang (1999) [47]	N/R	N/R	N/R	N/R	Median (range)<br>EF<br>IG: from 57.8 (39.5-64.8) to 69.5 (59.2-99.6)<br>by wk 6<br>p =0.001***<br><br>RVF<br>IG: from 57.7 (43.5-75.3) to 34.1(21.5-52.1)<br>by wk 6<br>p = 0.001***	N/R	N/R	N/R	N/R	N/R
Kan (2001) [43]	N/R	N/R	N/R	N/R	Median (range)<br>EF<br>IG: from 40 (36-44) to 65 (57-68)<br>(p =0.006**)<br>CG: small changes<br>by day 8<br>p <0.001***; MD 23%; 95% CI, 18.5%-28.5%<br>RVF	N/R	N/R	N/R	N/R	N/R

					IG: from 56 (51-59) to 40(31-49) (p = 0.008**) CG: small changes by dy 8					
Davies (2007) [41]	N/R	N/R	N/R	N/R	N/R	N/R	Median IG: 5.2 to 2 by wk 24	N/R	N/R	N/R
Jull (2009) [42]	No significant difference b/w IG and CG for time to healing P = .49	IG: 8(38%) CG:10 (53%) by wk 12	Mean change IG: 1.47cm^2 CG: -2.92cm^2 95% CI: 1.6 to 2.7cm^2 by wk 12 p = .08	N/R	Mean EF IG: from 74.3 to 102.1 CG: from 70.5 to 79.6 (p<0.05) by wk12<br><br>RVF IG: from 23.1 to 23.9 CG: from 21.1 to 30.9 (p=0.3) by wk12	N/R	N/R	1 or 2 adverse events were reported by:<br><br>IG: 19 (59%) CG: 3 (41%) (OR 1.32, 95% CI: 0.95 to 1.85)	N/R	N/R
Meagher (2012) [48]	No significant associatio n b/w the number of daily steps and time to healing	IG: 15(83%) CG 13(76%) by wk 12 *P*=.128	N/R	N/R	N/R	N/R	N/R	N/R	N/R	N/R

	p = .870									
Ahmed (2013) [40]	N/R	N/R	Means ± SD (cm^2)<br>IG:4.55±1.14<br>CG: 7.43±0.56<br>by dys 12<br>p ≤ .05	N/R	N/R	N/R	N/R	N/R	N/R	N/R
O'Brien (2013) [38]	N/R	IG: 50% CG: 40%<br>by wk 12<br>χ2 = 0·11, p = 0·74	IG: 77% CG: 45%<br>by wk 12<br>*P* = ·34.	N/R	Mean (SD)<br>EF:<br>IG: from 55 (9) to 66.55 (12) (p = .05)<br>CG: from 60.33 (14) to 61.53 (15)<br>by wk 12<br>($F_{1,5}$ = 6.7, p =0.05*)<br><br>RVF:<br>IG: from 44·92 (8) to 33.06(12) (p = 0·03)<br>CG: from 39.67 (13) to 38.45(15)<br>by wk 12<br>($F_{1,5}$ = 7.02, p =0.04*)	N/R	N/R	No adverse events	N/R	N/R
Sallam (2017) [39]	N/R	N/R	Mean ±SD (cm^2)<br>IG:8.8±3.8<br>CG:13.9±5.1<br>by wk 12	N/R	N/R	N/R	Mean ±SD<br>IG: 4.1±1.6 to 2.8±1.1<br>CG: 3.8±1.6 to 3.6±1.7<br>by wk 12	No adverse events	N/R	N/R
O'Brien (2017) [44]	N/R	IG: 77%<br>CG: 53%<br>By wk 12<br>p =0.09	N/R	N/R	N/R	Mean ±SD<br>PCS<br>IG:46±10·2<br>CG:43±8·9<br>by wk 12	N/R	N/R	N/R	N/R

						Median (range)<br>MCS<br>IG: 51 (16–65)<br>CG: 51 (32–66)<br>By wk 12				
Domingues (2018) [49]	N/R	N/R	IG had significant area reduction comparing to CG by dys 90<br>p =0.01	N/R	N/R	Significant difference was observed in QoL for IG comparing with CG by dys 90<br>p =0.03*	CG had worsened pain comparing with IG by dys 90<br>p =0.44	No adverse events	N/R	N/R
Klonizakis (2018) [51]	Median (range)<br>IG:13 wks (3.9–52)<br>CG:34.7 wks (4.3–52)<br>by mth 12	IG: 14/17 (83%)<br>CG: 12/20 (60%)<br>by mth 12	Median (range) (cm^2)<br>IG:0 (0–18.7)<br>CG: 0 (0–147)<br>by mth 12	IG: 2/17 (12%)<br>CG: 1/19 (5%)<br>by mth 12	N/R	Mean ±SD<br>EQ-5D-5L<br>IG: 0.7874±0.28<br>CG: 0.5825± 0.41<br>by mth 12<br><br>VEINES-QOL<br>IG: 67.23 ±29.86<br>CG: 52.46 ±34.81<br>by mth 12	Mean ±SD<br>IG: 24.44 ±27.3 to 7.9±22.8<br>CG: 30.95 ± 31.6 to 30.5±36.6<br>by mth 12	Increased exudate (n=2) by mth 12	Out-of-pocket (total) (£)<br>IG:1931.76<br>CG:3666.12<br><br>Mean per patient (£)<br>IG: 113.63<br>CG: 174.58<br><br>by mth 12	National Health Service (total) (£)<br>IG:13825.60<br>CG:48270.00<br><br>Mean per patient (£)<br>IG: 813.27<br>CG: 2298.57<br><br>by mth 12
Mutlak (2018) [45]	N/R	N/R	Median (range) (cm)<br>IG: 0.72 (1.17-0.12)<br>CG: 2.52 (3.34-0.63)	N/R	N/R	N/R	N/R	N/R	N/R	N/R

			by mth 3							
Nabil (2019) [50]	N/R	N/R	Mean ±SD<br>IG:1.6±0.62 (cm^3)<br>CG: 2.39±0.75 (cm^3)<br>(p =0.004**)<br>by wk 12	N/R	N/R	N/R	N/R	N/R	N/R	N/R
Jonker (2020) [37]	N/R	Heal/ Not heal<br>IG:10(67%)/5 (33%)<br>CG: 7(41%)/ 10(59%)<br>p=0.18<br>by wk 12	Median (IQR)<br>IG: 0 (3.23)<br>CG:0.12(0.88) p =0.73<br>by wk 12	N/R	N/R	N/R	IG: 3/10 to 0/10<br>CG: 4/10 to 2/10<br>by wk 12	Increased exudate (n=1) by mth 1	N/R	N/R
Kelechi (2020) [46]	N/R	N/R	N/R	N/R	N/R	Mean ±SD<br>PCS<br>FOOTFIT: 27.3 ± 9.6<br>FOOTFIT+ app: 39.0 ± 9.9<br>by wk 6<br><br>MCS<br>FOOTFIT: 57.9 ± 7.4<br>FOOTFIT+ app: 61.3 ± 4.8<br>by wk 6	N/R	N/R	N/R	N/R

Note: between, b/w; chi-square, χ2; confidence interval, CI; control group, CG; day(s), dy(s);degree of freedom, F; ejection fraction, EF; five-level EuroQol five-dimensional questionnaire, EQ-5D-5L; highly significant,***; intervention group, IG; mean deviation, MD; mental component

score, MCS; month(s), mth(s); not reported, N/R; odds ratio, OR; physical component score, PCS; residual volume fraction, RVF; significant, *; standard deviation, SD; very significant, **; week(s), wk(s); venous insufficiency epidemiological and economic study on quality of life, VEINES-QoL.
